# Vancomycin-associated acute kidney injury in Hong Kong in 2012–2016

**DOI:** 10.1186/s12882-020-1704-4

**Published:** 2020-02-03

**Authors:** Xuzhen Qin, Man-Fung Tsoi, Xinyu Zhao, Lin Zhang, Zhihong Qi, Bernard M. Y. Cheung

**Affiliations:** 1 Department of Laboratory Medicine, Chinese Academy of Medical Sciences & Peking Union Medical College Hospital, Beijing, China; 2 Department of Medicine, The University of Hong Kong, Hong Kong, China; 3 Department of epidemiology and health statistics, Institute of basic medicine, Peking Union Medical College, Beijing, China; 4 State Key Laboratory of Pharmaceutical Biotechnology, The University of Hong Kong, Hong Kong, China

**Keywords:** Vancomycin, Acute kidney injury

## Abstract

**Background:**

To study the incidence of vancomycin-associated acute kidney injury (VA-AKI) in Hong Kong and identify risk factors for VA-AKI.

**Method:**

Patients with vancomycin prescription and blood level measurement in 2012–2016 were identified using the Hong Kong Hospital Authority Clinical Data Analysis and Reporting System. Acute kidney injury was defined using KDIGO criteria. Patients without creatinine measurements, steady-state trough vancomycin level or who had vancomycin treatment < 3 days were excluded. Results were analyzed using SPSS version 22.0. Logistic regression was used to identify the predictors for VA-AKI. Odds ratio and 95% confidence interval were estimated.

**Results:**

One thousand four hundred fifty patients were identified as VA-AKI from 12,758 records in Hong Kong in 2012–2016. The incidence was respectively 10.6, 10.9, 11.3, 12.2, 11.2% from 2012 to 2016. The incidence of VA-AKI was 16.3, 12.2, 11.3 and 6.2% in patients aged 1–12, 12–60, elderly aged > 60 and newborn and infants, respectively. Baseline creatinine, serum trough vancomycin level, systematic disease history including respiratory failure, hypertension, congestive heart failure, chronic renal failure, anemia and type II diabetes, and concomitant diuretics, piperacillin-tazobactam (PTZ) and meropenem prescription were significantly higher in VA-AKI patients older than 12 years. Logistic regression showed that older age group, higher baseline creatinine, serum trough vancomycin level, respiratory failure, chronic renal failure and congestive heart failure, concomitant diuretics, PTZ and meropenem prescription, and longer hospital stay were all associated with increased risk of VA-AKI.

**Conclusion:**

The incidence of VA-AKI in Hong Kong is low but shows no decline. Patients with higher baseline creatinine, multi-organ diseases and multiple drugs administration should have their vancomycin level monitored to decrease the risk of VA-AKI.

## Background

Methicillin-resistant *Staphylococcus aureus* (MRSA) has gained considerable attention due to its high morbidity, limited effective treatments available and increased incidence [1]. Vancomycin has been recommended as the first line treatment for MRSA infections by American, European, and Chinese guidelines for infectious diseases control and management control for antibiotics [2–5]. However, the significant nephrotoxicity limits the use of vancomycin in daily clinical practice. It has been reported the incidence of VA-AKI ranges from 0% to over 40% [6].

AKI is one of the examples of nephrotoxicity that accounts for about 1.7 million deaths every year worldwide. The consequences of AKI can be severe, ranging from hospitalization to life-long dialysis and transplantation. From a recent meta-analysis, the pooled incidence of AKI was 19.4% in eastern Asia [7]. Drugs are the third main cause of AKI [8]. The prognosis in drug-associated AKI is poor, especially in critically ill patients.

In Hong Kong, there is limited published literature on the incidence of drug-induced nephrotoxicity. A previous study reported an incidence of 23.1% for VA-AKI in a hospital [9]. A systematic population-wide study is clearly needed, in view of the serious implications of this potentially preventable and reversible condition. Therefore, we investigated the incidence of VA-AKI in Hong Kong from 2012 to 2016, characterized patients with VA-AKI as well as identified predictors for the development of VA-AKI.

## Methods

### Study design and data sources

A retrospective study was conducted using Clinical Data Analysis and Report System (CDARS), a database managed by the Hong Kong Hospital Authority (HA). HA is the sole public hospital service provider in Hong Kong. More than 90% of Hong Kong residents use the public hospital services [10]. CDARS captures patient records in Hong Kong in all public hospitals and outpatient clinics since 1993. This database provides a convenient platform for pharmaco-epidemiological studies [11].

All patients’ records with vancomycin prescription and measurement of serum drug level from January 2012 to December 2016 in Hong Kong were retrieved from CDARS. Demographics, laboratory parameters, concomitant medications and co-morbidities were collected as the covariates. Patients with vancomycin prescription and steady-state serum trough vancomycin level were included as the exposure status. Patients who had no baseline and follow-up concentration of creatinine within 48 h, vancomycin treatment < 3 days or steady state serum trough vancomycin level were excluded. Trough concentrations were defined as the results from the samples taken within 1 h of the next due dose. The first trough concentration was measured after 3 days of vancomycin administration.

### Definition of AKI

With the use of Kidney Disease Improving Global Outcomes (KDIGO) criteria [12], patients with AKI were identified as increase in serum creatinine (SCr) by 0.3 mg/dL or more within 48 h, or as a 1.5~1.9 times increase in SCr from baseline. These patients were at least of stage I in severity. Stage II was defined as 2~2.9 times increase in SCr from baseline. Stage III was defined as 3 times increase in SCr from baseline or increase in SCr to ≥4 mg/dL. Vancomycin-associated AKI was the main outcome for the study. Patients without VA-AKI were classified as non-VA-AKI group.

### Statistical analysis

IBM SPSS statistics version 22.0 (IBM SPSS Statistics, Armonk, NY, USA) was used for data analysis. Descriptive statistics were expressed as frequency for categorical data or median (interquartile range) for continuous variables. With reference to a vancomycin therapeutic guideline [5], vancomycin concentration was categorized into 4 groups: < 10.0 mg/L, 10.0~15.0 mg/L, 15.0~20.0 mg/L, > 20.0 mg/L. Continuous variables were compared among groups using one-way ANOVA and Kruskal-Wallis tests as appropriate. Categorical variables were compared among groups using Pearson Chi-square test. Logistic regression was used to estimate odd ratios (ORs) and 95% confidence interval (CI). Firstly, univariate logistic models were used to explore the relationship between VA-AKI and risk factors, including gender, age, route of administration, baseline creatinine, tough vancomycin level, concomitant drugs, length of stay and death in hospital. Secondly, to determine whether there was significant collinearity between continuous variables, collinearity diagnostics were performed using variance inflation factor (VIF) and tolerance values. If tolerance value is close to zero or VIF is greater than 10, there may be multi-collinearity. Thirdly, four models adjusted by different confounders were applied to analyse the possible predicators. Model 1: adjusted for demographics (age, sex); Model 2: additionally adjusted for comorbidities; Model 3: additionally adjusted for medications; Model 4: additionally adjusted for baseline creatinine and length of stay. Predictor variables were entered if *P* < 0.05 and removed if *P* > 0.10 in the stepwise model. A two-tailed P < 0·05 was considered statistically significant. A two-tailed P < 0.05 will be considered statistically significant.

## Results

**Fig. 1:**
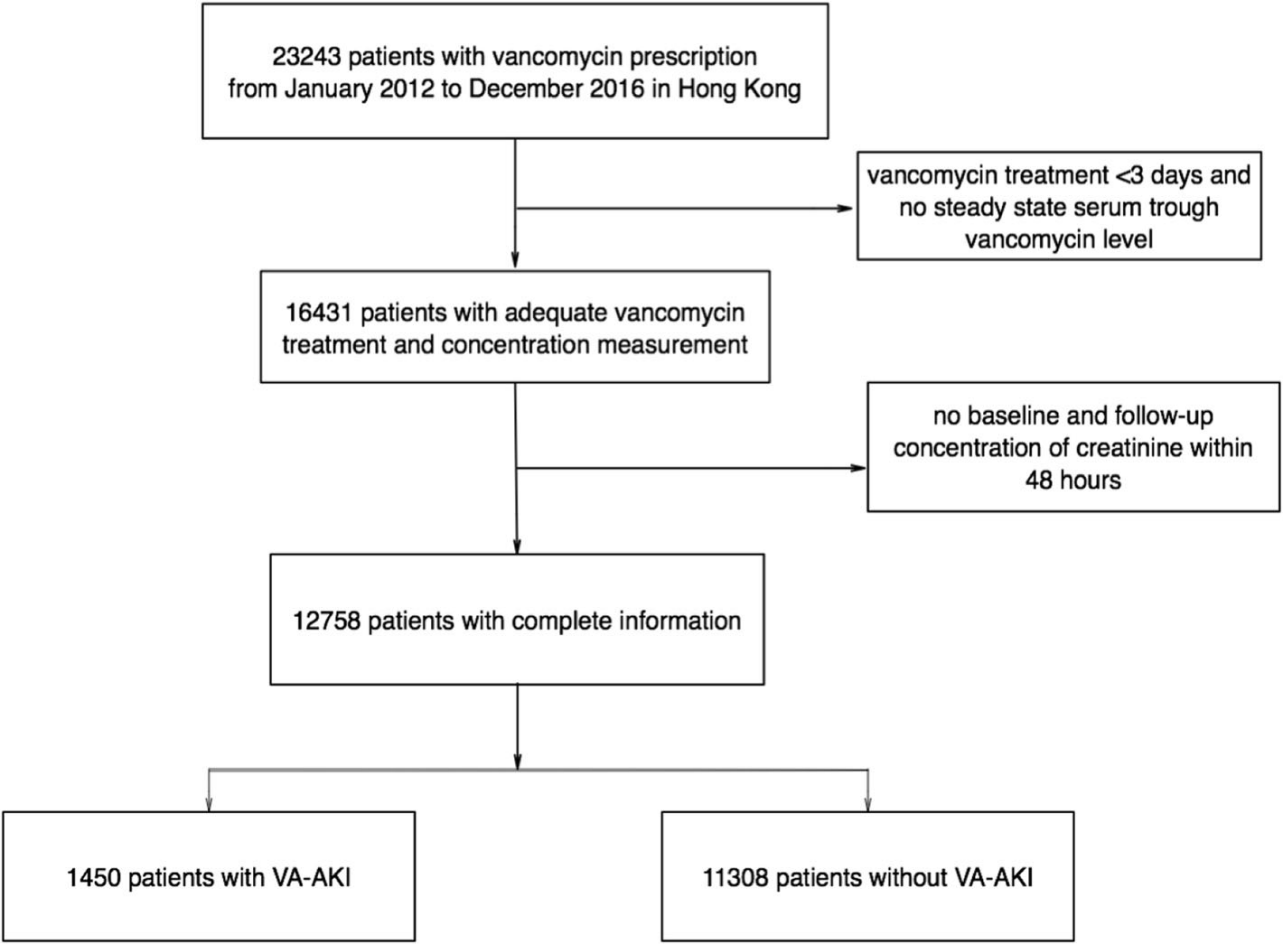
A flow diagram for inclusion of study samples

A total of 12,758 patients were included in this study from 2012 to 2016 in Hong Kong (Fig. 1). The median age of patients with VA-AKI was 72 years old, and 64.2% of cases were male. The prescription of vancomycin increased gradually and doubled in 2016 compared with 2012. The incidence of VA-AKI remained at approximately 11.4% (Table 1).

**Table 1:** Incidence and demographic characters of patients with VA-AKI in Hong Kong from 2012 to 2016

	2012	2013	2014	2015	2016
Annual admissions	1,466,788	1,508,489	1,532,926	1,592,148	1,629,346
Vancomycin prescription	3310	3510	4132	5597	6694
Enrolled patients	1542	1709	2446	3374	3687
Male	63.1%	62.5%	62.9%	63.7%	64.0%
Female	36.9%	37.5%	37.1%	36.3%	36.0%
Age	71.7 (52.8, 83.0)	71.6 (55.8, 81.9)	73.8 (58.5, 83.4)	71.8 (58.1, 83.5)	76.5 (62.1, 85.7)
VA-AKI cases	164	186	276	410	414
Incidence of VA-AKI	10.6%	10.9%	11.3%	12.2%	11.2%

**Fig. 2:**
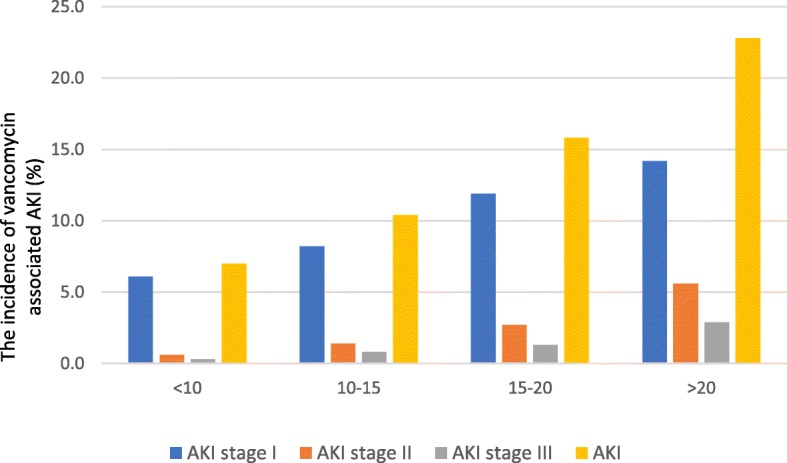
The incidence of vancomycin associated AKI stratified by vancomycin level (mg/L) and AKI stage

Table 2 shows the characteristics of patients with VA-AKI stratified by age group. The incidence of VA-AKI in patients aged 1 to 12 was the highest (16.3%), following patients aged 12 to 60 (12.2%), elderly aged over 60 (11.3%) and newborn and infants (6.2%). The median age of VA-AKI patients was slightly younger than patients without VA-AKI in elderly group, yet older than patients without VA-AKI in patients less than 60 years old. There was no significant gender difference between VA-AKI and non-VA-AKI groups in all age groups. The main route of administration was injection and parenteral, with several cases of oral, eye drop and intraperitoneal route. The incidence of VA-AKI was distinctively higher in newborn and infants receiving parenteral administration (*P* < 0.001). The baseline creatinine and trough vancomycin concentration in the VA-AKI group were significantly higher than non-VA-AKI group in patients older than 12 years old (*P* < 0.001). However, there were no significant difference in patients aged under 12. Along with the increase of trough vancomycin level, the incidence of VA-AKI either in cumulative incidence or in each AKI stage showed a significantly upward trend (*P* < 0.001) (Fig. 2). Compared to non-VA-AKI group, more patients with VA-AKI aged over 12 had comorbidities including respiratory failure, essential hypertension, congestive heart failure, chronic renal failure, anaemia and type II DM. There were more comorbidities in VA-AKI patients between 12 to 60 years old compared with non-VA-AKI group in the same age group. Patients with VA-AKI aged over 12 were more likely to receive diuretics, piperacillin-tazobactam (PTZ) and meropenem, while more angiotensin converting enzyme inhibitors (ACEI) prescripts were found in patients with VA-AKI from 1 to 12 years old, more ACEI, angiotensin II receptor blocker (ARB), non-steroidal anti-inflammatory drugs (NSAID) prescripts in VA-AKI group from 12 to 60 years old, and more aminoglycoside and amphotericin B prescripts in VA-AKI patients over 60 years old. Patients over 60 years old with VA-AKI were hospitalized for longer duration. VA-AKI group had a significantly higher mortality in hospital compared with non-VA-AKI group.

**Table 2:** Comparison of clinical characteristics in VA-AKI and non-VA-AKI patients stratified by age groups

	New-borns and infants			Children 1 to 12 years of age			Adolescents above 12 years of age and adults			Elderly patients aged over 60 years		
	Non-VA-AKI	VA-AKI	*P* value	Non-VA-AKI	VA-AKI	P value	Non-VA-AKI	VA-AKI	P value	Non-VA-AKI	VA-AKI	*P* value
N	406 (93.8%)	27 (6.2%)	–	123 (83.7%)	24 (16.3%)	–	2523 (87.8%)	350 (12.2%)	–	8256 (88.7%)	1049 (11.3%)	–
Age	0.0 (0.0, 0.1)	0.0 (0.0, 0.2)	0.034	4.5 (2.7, 7.7)	4.8 (1.4, 6.3)	0.277	50.6 (41.1, 56.0)	52.2 (43.8, 56.6)	0.037	79.9 (71.1, 86.4)	78.1 (69.0, 84.6)	< 0.001
Male	59.1%	55.6%	0.840	52.8%	70.8%	0.120	65.0%	66.6%	0.590	63.1%	63.4%	0.865
Strains of bacteria												
Streptococcus	1	0	0.594	5	0	0.231	51	3	0.039	82	14	0.104
Enterococcus	11	1		1	0		10	1		106	20	
Staphylococcus	55	2		9	2		158	27		1061	145	
Route of Administration												
injection	98.0%	85.2%	< 0.001	94.3%	95.8%	0.763	82.1%	85.7%	0.049	76.2%	78.6%	0.279
parenteral	2.0%	14.8%		5.7%	4.2%		17.6%	13.7%		23.5%	21.1%	
oral	–	–		–	–		0.2%	0.3%		0.2%	0.2%	
Eye drop	–	–		–	–		0.0%	0.3%		0.0%	0.1%	
Intraperitoneal	–	–		–	–		0.1%	0.0%		0.0%	0.2%	
Chemistry Profile												
Baseline Creatinine (μmol/L)	58.2 (41.0, 74.0)	44.0 (20.0, 69.0)	0.060	36.0 (28.4, 46.0)	31.5 (23.8, 46.3)	0.256	66.0 (51.0, 94.0)	112.2 (68.0, 269.0)	< 0.001	79.0 (58.3, 122.0)	111.0 (72.7, 196.5)	< 0.001
Tough Vancomycin (mg/L)	7.2 (5.0, 10.8)	9.7 (5.8, 14.7)	0.075	5.2 (3.3, 9.2)	11.7 (5.7, 19.7)	< 0.001	7.8 (5.1, 12.5)	13.8 (8.6, 20.5)	< 0.001	11.3 (7.8, 16.2)	15.0 (9.9, 21.1)	< 0.001
Comorbidities												
Pneumonia	7.1%	11.1%	0.439	41.5%	25.0%	0.171	21.6%	28.0%	0.009	38.5%	37.9%	0.723
Respiratory failure	9.4%	11.1%	0.733	12.2%	25.0%	0.115	20.4%	26.6%	0.010	19.4%	24.1%	< 0.001
Essential hypertension	0.2%	0.0%	1.000	0.8%	0.0%	1.000	17.1%	28.0%	< 0.001	50.3%	53.6%	0.048
Congestive heart failure	2.0%	0.0%	1.000	1.6%	4.2%	0.417	7.3%	18.0%	< 0.001	19.3%	24.9%	< 0.001
Urinary tract infection	4.9%	3.7%	1.000	5.7%	8.3%	0.641	15.4%	15.4%	1.000	38.4%	34.5%	0.015
Chronic renal failure	0.2%	0.0%	1.000	0.8%	8.3%	0.069	7.3%	24.0%	< 0.001	6.5%	16.0%	< 0.001
Fever	3.9%	7.4%	0.311	34.1%	29.2%	0.814	25.3%	24.9%	0.896	36.1%	34.9%	0.429
Sepsis	7.1%	3.7%	1.000	14.6%	12.5%	1.000	13.4%	15.4%	0.318	21.3%	21.4%	0.938
Anaemia	12.3%	11.1%	1.000	18.7%	20.8%	0.781	21.4%	27.7%	0.009	25.8%	29.9%	0.004
Type II DM	–	–	–	–	–	–	12.1%	18.9%	0.001	27.0%	30.3%	0.024
Number of comorbidities												
0	66.0%	55.6%	0.379	30.1%	37.5%	0.732	29.9%	19.4%	< 0.001	10.2%	9.6%	0.114
1	23.2%	37.0%		31.7%	20.8%		24.9%	18.9%		15.0%	12.4%	
2	7.9%	3.7%		21.1%	25.0%		19.3%	20.3%		18.2%	18.9%	
> 3	3.0%	3.7%		17.1%	16.7%		25.8%	41.4%		56.5%	59.1%	
Concomitant drugs												
Inotropic agents	2.5%	0.0%	1.000	4.1%	4.2%	1.000	4.5%	8.8%	0.002	11.4%	12.8%	0.184
Diuretics	71.2%	74.1%	0.829	48.0%	62.5%	0.265	54.2%	72.7%	< 0.001	64.2%	73.5%	< 0.001
ACEI	2.7%	7.4%	0.191	6.5%	33.3%	0.001	19.4%	30.6%	< 0.001	32.5%	34.2%	0.285
ARB	–	–	–	0.8%	0.0%	1.000	5.2%	11.5%	< 0.001	7.5%	9.0%	0.079
PTZ	12.3%	7.4%	0.758	26.8%	29.2%	0.806	62.0%	73.3%	< 0.001	62.0%	67.9%	< 0.001
Meropenem	53.0%	44.4%	0.430	41.5%	66.7%	0.027	51.2%	59.4%	0.006	42.5%	47.6%	0.002
Aminoglycoside	54.4%	33.3%	0.045	59.3%	45.8%	0.262	52.7%	50.9%	0.557	45.6%	49.4%	0.024
Cefepime	89.4%	100.0%	0.094	84.6%	70.8%	0.141	69.3%	72.7%	0.225	64.6%	66.2%	0.317
Amphotericin B	6.9%	11.1%	0.429	8.1%	20.8%	0.072	8.6%	8.5%	1.000	2.1%	3.2%	0.019
NSAIDs	1.2%	0.0%	1.000	31.7%	29.2%	1.000	30.1%	21.8%	0.002	15.7%	14.7%	0.367
Number of concomitant drugs												
0	2.0%	0.0%	0.685	0.8%	0.0%	0.738	2.7%	1.8%	< 0.001	2.9%	2.0%	< 0.001
1	10.1%	7.4%		15.4%	12.5%		8.0%	3.0%		8.2%	4.9%	
2	24.9%	33.3%		24.4%	16.7%		16.9%	7.6%		16.4%	13.1%	
> 3	63.1%	59.3%		59.3%	70.8%		72.4%	87.6%		72.5%	80.0%	
Length of Stay	85.0 (47.8, 122.0)	107.0 (31.0, 155.0)	0.608	36.5 (21.0, 61.0)	39.5 (12.3, 87.5)	0.778	50.0 (27.0, 87.0)	44.0 (25.0, 85.0)	0.249	40.0 (23.0, 67.0)	33.0 (20.0, 58.0)	< 0.001
Death in hospital	8.4%	18.5%	0.075	9.8%	25.0%	0.037	16.8%	31.4%	< 0.001	28.4%	51.5%	< 0.001

*Abbreviations: Type II DM* Type II diabetes metillus, *ACEI* angiotension converting enzyme inhibitors, *ARB* angiotensin II receptor blocker, *PTZ* piperacillin-tazobactam, *NSAIDs* non-steroidal antiinflammatory drugs

Logistic analysis shows the possible risk factors associated with VA-AKI (Table 3). Using both univariate logistic model and adjusted logistic models showed that older age, baseline creatinine, trough vancomycin level, the comorbidities including essential hypertension, anaemia, respiratory failure, type II DM, chronic renal failure and congestive heart failure, concomitant drugs including diuretics, ACEI, ARB, PTZ, and meropenem were all associated with higher risk for the development of VA-AKI. Regarding collinearity, the highest VIF and the lowest tolerance values were 1.016 and 0.87, respectively, which suggests the absence of significant collinearity. Compared with newborns and infants, older age groups had lower ORs for development of VA-AKI (OR of children 1 to 12 years of age: 2.93 (95%CI [1.63, 5.27], OR of adults and adolescents above 12 years of age: 2.09 (95%CI [1.39, 3.13], OR of elderly patients: 1.91 (95%CI [1.29, 2.84], respectively). Most of these predicators had positive relationships with VA-AKI, yet, urinary tract infection had a negative relationship with VA-AKI (OR: 0.75 (95%CI [0.66, 0.86]). After adjusted for gender, age, comorbidities, concomitant drugs, baseline creatinine and length of stay in various models with increasing confounders, vancomycin, especially vancomycin concentration over 10 were still significant predicators for VA-AKI. Compared to the vancomycin level less than 10.0 mg/L group, higher vancomycin level had higher OR for VA-AKI onset in univariate logistic model (OR of 10.0~15.0 level: 1.55 (95%CI [1.34, 1.81], OR of 15.1~20.0 level: 2.50 (95%CI [2.13, 2.93], OR of > 20.0 level: 3.89 (95%CI [3.34, 4.53], respectively).

**Table 3:** Predicators for VA-AKI in Hong Kong

	OR	Model 1	Model 2	Model 3	Model 4
Male	0.96 (0.86, 1.08)	–	–	–	–
Age					
Newborn and infants	Reference	–	–	–	–
Children 1 to 12 years of age	2.93 (1.63, 5.27)*	–	–	–	–
Adults and adolescents above 12 years of age	2.09 (1.39, 3.13)*	–	–	–	–
Elderly patients	1.91 (1.29, 2.84)	–	–	–	–
Chemistry profile					
Baseline creatinine	1.00 (1.00, 1.00)*	1.00 (1.00, 1.00)*	1.00 (1.00, 1.00)*	1.00 (1.00, 1.00)*	–
Trough vancomycin level	1.04 (1.04, 1.05)*	1.05 (1.04, 1.05)*	1.04(1.04, 1.05)*	1.04 (1.04,1.05)*	1.04 (1.04,1.05)*
Classification of vancomycin level (mg/L)					
< 10.0	Reference	Reference	Reference	Reference	Reference
10.0~15.0	1.55 (1.34, 1.81)*	1.68 (1.44, 1.96)*	1.63 (1.39, 1.92)*	1.62 (1.38,1.89)*	1.64 (1.40,1.93)*
15.1~20.0	2.50 (2.13, 2.93)*	2.75 (2.33, 3.24)*	2.62 (2.21, 3.11)*	2.61 (2.20,3.08)*	2.62 (2.21,3.11)*
> 20.0	3.89 (3.34, 4.53)*	4.29 (3.67, 5.02)*	3.93 (3.34, 4.63)*	4.05 (3.45,4.77)*	4.07 (3.46,4.79)*
Comorbidities					
Pneumonia	1.06 (0.94, 1.19)	1.07 (0.96, 1.21)	–	–	–
Essential hypertension	1.23 (1.10, 1.38)*	1.33 (1.18, 1.50)*	–		–
Urinary tract infection	0.87 (0.77, 0.98)#	0.88 (0.77, 1.00)	–	–	–
Fever	0.97 (0.86, 1.09)	0.98 (0.87, 1.11)	–	–	–
Anemia	1.29 (1.14, 1.45)*	1.30 (1.15, 1.48)*	–	–	–
Respiratory failure	1.39 (1.22, 1.58)*	1.39 (1.22, 1.58)*	–	–	–
Type II DM	1.25 (1.10, 1.41)*	1.28 (1.13, 1.46)*	–	–	–
Sepsis	1.03 (0.90, 1.19)	1.05 (0.91, 1.20)	–		–
Chronic renal failure	3.17 (2.72, 3.70)*	3.18 (2.72, 3.71)*	–	–	–
Congestive heart failure	1.56 (1.37, 1.79)*	1.63 (1.42, 1.87)*	–	–	–
Concomitant drugs					
Inotropic agents	1.26 (1.05, 1.50)#	1.28 (1.07, 1.53)#	1.19 (0.99,1.43)	–	–
Diuretics	1.71 (1.51, 1.94)*	1.72 (1.51, 1.95)*	1.49 (1.31,1.70)*		–
ACEI	1.25 (1.11, 1.41)*	1.27 (1.12, 1.43)*	1.11 (0.98,1.27)	–	–
ARB	1.46 (1.20, 1.77)*	1.47 (1.21, 1.79)*	1.15 (0.94,1.42)	–	–
PTZ	1.39 (1.24, 1.57)*	1.41 (1.25, 1.59)*	1.39 (1.23,1.57)*	–	–
Meropenem	1.29 (1.16, 1.45)*	1.29 (1.15, 1.44)*	1.23 (1.10,1.39)*	–	–
Aminoglycoside	1.71 (0.96, 1.22)	1.07 (0.95, 1.21)#	1.05 (0.94,1.18)	–	–
Cefepime	1.31 (1.01, 1.71)	1.29 (0.99, 1.69)	1.04 (0.92,1.18)		–
Amphotericin B	0.84 (0.73, 0.98)#	0.84 (0.72, 0.97)	1.24 (0.95,1.63)	–	–
NSAIDs	1.00 (1.00, 1.00)#	1.00 (1.00, 1.00)	0.93 (0.80,1.09)	–	–
Length of Stay	0.97 (0.86, 1.09)	0.98 (0.87, 1.11)	1.00 (1.00,1.00)	1.00 (1.00,1.00)#	–

Data is expressed as odds ratio (95% confidence interval)

-Not available or not in the equation

Model 1: adjusted for age and gender

Model 2: additionally, adjusted for comorbidities;

Model 3: additionally, adjusted for medications;

Model 4: additionally, adjusted for baseline creatinine and length of stay

*p < 0.001 #*p* < 0.05

## Discussion

In this study involving 12,578 patients on vancomycin, the incidence of VA-AKI was 11.4% in Hong Kong in 2012–2016. Compared with non-VA-AKI group, VA-AKI patients older than 12 years had significant higher level or frequency in the baseline creatinine, trough vancomycin level, systematic disease history including respiratory failure, essential hypertension, congestive heart failure, chronic renal failure, anaemia and type II DM, and concomitant drugs usage including diuretics, PTZ and meropenem. VA-AKI group had a significantly higher mortality in hospital compared with non-VA-AKI group. The incidence of VA-AKI increased with increased vancomycin level. Stepwise logistic regression showed that older age group, higher vancomycin level, comorbidities including respiratory failure, chronic renal failure and congestive heart failure, concomitant drugs including diuretics, PTZ and meropenem and length of stay were all independent risk factors for the development of VA-AKI.

This study is the first large scale investigation of VA-AKI in Hong Kong. In previous studies, the incidence of VA-AKI in Chinese varied from 2.4 to 45% [9, 13–16] (Additional file 1: Table S1). The difference of estimated incidence is due to different populations, sample sizes, inclusion criteria and AKI definition. In USA, the incidence of VA-AKI declined from 42.6% in 2007 to 14.1% in 2017 [17, 18]. This might be due to doctors’ awareness of the toxicity of vancomycin and improvements in vancomycin purity. Our study reports a lower incidence of VA-AKI than the previous study [9], since the large database is less prone to publication and other biases.

In the literature, nephrotoxicity occurs in 1–9% of neonates receiving currently recommended doses, and 6–14% of paediatric patients [19–21]. Since these studies were all based on different population, the incidence of VA-AKI cannot be compared. Our study compared the incidence of VA-AKI in different age group based on the same population. By age group, the order of incidence from high to low was children from 1 to 12 years old (16.3%), adolescents above 12 years of age and adults (12.2%), elderly patients aged over 60 years (11.3%), new-borns and infants (6.2%). The characters of young patients with VA-AKI were different from elderly patients. The median of trough vancomycin in patients under 60 years old were all lower than 10 mg/L, yet the incidence of VA-AKI still accounted for a sizeable proportion. In new-borns and infants receiving parenteral administration the incidence of VA-AKI was distinctively higher.

There have been studies on the risk factors for VA-AKI in the past [22–24]. Like previous studies, a positive correlation between VA-AKI and increasing vancomycin trough concentrations and baseline serum creatinine was found in this study. The association between age and VA-AKI was controversial, mostly with a positive correlation [24]. While some studies explored after controlling for comorbid condition, medications and renal function, age is not associated with AKI [25]. In this study, we found age was correlated with VA-AKI, yet compared with new-borns and infants age group, older age group had less OR for development of VA-AKI. Concomitant administration of diuretics, PTZ and meropenem could increase the incidence of nephrotoxicity [22, 26, 27], except concomitant NSAIDs seems to have a protective role for VA-AKI. In each age group, VA-AKI incidence was lower in patients with concomitant NSAIDs. We confirmed that concomitant chronic renal failure, congestive heart failure and respiratory failure increased the risk for VA-AKI, as in a previous study in China [15]. Although vancomycin administrated by eye-drops, oral and intraperitoneal has low bioavailability, a previous study reported AKI development was related with the usage of topical fortified gentamicin and vancomycin eyedrops [28]. Similarly, serum concentration of vancomycin could also be observed in VA-AKI group in different routes of administration in this study. The incidence and mechanism of VA-AKI in uncommon routes of administration remains uncertain, yet the possible risk for AKI development should be noticed.

Several explanations have been put forth on the cause of VA-AKI, including impurities in vancomycin [29], critically-ill patients with complications and on multiple drugs, and lack of suitable control patients. A randomized controlled trial was conducted to identify the relationship between vancomycin and the development of AKI. Patients were randomized to continuation of dose-optimized vancomycin or early switch to an alternative antimicrobial agent. The incidence of AKI in both groups was similar (32.7 to 31.4% [*P* = 0.89]) [30], but this was a small study of 103 patients in a single center.

The strength of this study was that it was population-based and territory-wide, and used a very large database. In the limited literature, the incidence of AKI in Hong Kong was reported as 11.7% [31], similar to the incidence of VA-AKI in our study. It provides good evidence that dosing and monitoring practices of vancomycin in Hong Kong are sound. However, there were limitations. Since it was an observational study, causation cannot be proven. Patients with VA-AKI could have other reasons for AKI, such as comorbidities, hypotension, sepsis and concomitant drugs. It is distinctly difficult to determine the actual risk factors of VA-AKI.

## Conclusion

In summary, the incidence of VA-AKI in Hong Kong is low but shows no decline. As there is increased usage of vancomycin to combat MRSA, there should be increased awareness of VA-AKI. Patients with higher baseline creatinine, chronic diseases and multiple drugs are at risk of VA-AKI and should have their vancomycin level monitored. Our findings facilitate the development of strategies and guidelines to prevent vancomycin-associated AKI.

## Supplementary information

**Additional file 1: Table S1.** Literature review of incidence of VA-AKI in Chinese. The key points of previous publications regarding to VA-AKI in Chinese were summarized including the region, patients, definition of AKI, incidence of VA-AKI and references.

## Data Availability

The datasets used and/or analysed during the current study available from CDARS on reasonable request.

## Abbreviations

ACEI: Angiotension converting enzyme inhibitors
ARB: Angiotensin II receptor blocker
CDARS: Clinical Data Analysis and Report System
CI: Confidence interval
HA: Hong Kong Hospital Authority
KDIGO: Kidney Disease Improving Global Outcomes
MRSA: Methicillin-resistant *Staphylococcus aureus*
NSAID: Non-steroidal anti-inflammatory drugs
ORs: Odd ratios
PTZ: Qpiperacillin-tazobactam
SCr: Serum creatinine
VA-AKI: Vancomycin-associated acute kidney injury
