# Supplementary material for: Vancomycin-associated acute kidney injury in Hong Kong in 2012–2016
Source: BMC Nephrol. 2020 Feb 3;21:41. doi: 10.1186/s12882-020-1704-4 (PMC6998253; doi:10.1186/s12882-020-1704-4)
Supplement: Supplementary file 1 — Additional file 1: Table S1. Literature review of incidence of VA-AKI in Chinese. The key points of previous publications regarding to VA-AKI in Chinese were summarized including the region, patients, definition of AKI, incidence of VA-AKI and references. [file 12882_2020_1704_MOESM1_ESM.docx]

**Additional file 1: Table S1. Literature review of incidence of VA-AKI in Chinese**

| Region | Patients | Definition of AKI | Incidence | Literature |
| --- | --- | --- | --- | --- |
| Mainland | Adult | SCr >0.5 mg/dl (44.2 mmol/l), or an increase in SCr >50% from the baseline level for at least 2 days | 15.6% | Dong MH, et al. Int J Infect Dis. (2015) |
| Mainland | Children | Scr increase ≥44.2 lmol/L or a ≥50% increase in baseline Scr for at least two consecutive days | 2.4% | Wei WX, et al. J Clin Pharm Ther. (2016) |
| Mainland | Elderly | either as an increase in SCr level of 0.5 mg/dl or as a 1.5–2fold increase in SCr from baseline | 29% | Liu Y, et al. Pharmacology. (2015) |
| Taiwan | Inpatients | RIFLE criteria ‘risk’ (R), ‘injury’ (I), ‘failure’ (F) | 45% | Shen WC, et al. Nephrology (2011) |
| Hong Kong | Adult | 0.5 mg/dL or ≥50% from the baseline serum creatinine level in two consecutive laboratory tests. | 23.1% | You JH, et al. Int J Antimicrob Agents. (2011) |
